# 729. I Can CPO Clearing Now, the Iso is Gone

**DOI:** 10.1093/ofid/ofad500.790

**Published:** 2023-11-27

**Authors:** Danielle Schwartz, Kailee Trepanier, Ellina Lytvyak, Michael Zakhary, Joseph Kim, Uma Chandran

**Affiliations:** University of Alberta, Edmonton, Alberta, Canada; University of Alberta Faculty of Medicine and Dentistry, Edmonton, Alberta, Canada; University of Alberta, Edmonton, Alberta, Canada; University of Alberta, Edmonton, Alberta, Canada; University of Calgary, Calgary, Alberta, Canada; University of Alberta, Edmonton, Alberta, Canada

## Abstract

**Background:**

There is little research or guidance about clearing patients who are colonized with carbapenemase-producing organisms (CPO). The use of isolation precautions to reduce transmission needs to be balanced with the potential negative impacts of isolation on patient experience, quality of care and non-infection patient safety and outcome measures. Our objective was to develop and implement a standardized provincial protocol for clearing patients of their CPO-positive status.

**Methods:**

PubMed, Web of Science, EMBASE and Cochrane Library were searched from January 2000 to February 2023. Randomized control trials, retrospective and prospective cohort studies, cross-sectional studies and case series were included. This information was used to create a CPO clearing protocol for use by Alberta Health Services (AHS) Infection Prevention and Control (IPC). The protocol will be monitored through the provincial surveillance system (ProvSurv) to guide future changes.

**Results:**

The literature showed that although CPO colonization can be prolonged (more than 12 months), a significant number of patients tested CPO-negative by 3 months since the last positive test. In addition, one-third of patients with 1 or 2 negative swabs subsequently tested CPO-positive. Therefore, the proposed provincial guidance is to re-screen patients 3 months after their last CPO-positive result. Three negative sets (without antibiotic pressure) done at least one week apart are required to clear a CPO flag; where a negative set includes a rectal swab or stool culture, cultures of previously positive sites if still clinically relevant, and additional currently relevant sites.

**Conclusion:**

This project aimed to create a standardized provincial protocol for clearing patients of their CPO-positive status to decrease the duration of isolation, while also balancing resource management and patient-centred care. After implementation, next steps include evaluation by monitoring compliance and examining specific CPO-related indicators. There is potential for use by other healthcare institutions and organizations.

**Disclosures:**

**All Authors**: No reported disclosures

